# Solution Structures of the Acyl Carrier Protein Domain from the Highly Reducing Type I Iterative Polyketide Synthase CalE8

**DOI:** 10.1371/journal.pone.0020549

**Published:** 2011-06-02

**Authors:** Jackwee Lim, Rong Kong, Elavazhagan Murugan, Chun Loong Ho, Zhao-Xun Liang, Daiwen Yang

**Affiliations:** 1 Department of Biological Sciences, National University of Singapore, Singapore, Singapore; 2 School of Biological Sciences, Nanyang Technological University, Singapore, Singapore; Consejo Superior de Investigaciones Cientificas, Spain

## Abstract

Biosynthesis of the enediyne natural product calicheamicins γ_1_^I^ in *Micromonospora echinospora ssp. calichensis* is initiated by the iterative polyketide synthase (PKS) CalE8. Recent studies showed that CalE8 produces highly conjugated polyenes as potential biosynthetic intermediates and thus belongs to a family of highly-reducing (HR) type I iterative PKSs. We have determined the NMR structure of the ACP domain (*me*ACP) of CalE8, which represents the first structure of a HR type I iterative PKS ACP domain. Featured by a distinct hydrophobic patch and a glutamate-residue rich acidic patch, *me*ACP adopts a twisted three-helix bundle structure rather than the canonical four-helix bundle structure. The so-called ‘recognition helix’ (*α*2) of *me*ACP is less negatively charged than the typical type II ACPs. Although loop-2 exhibits greater conformational mobility than other regions of the protein with a missing short helix that can be observed in most ACPs, two bulky non-polar residues (Met^992^, Phe^996^) from loop-2 packed against the hydrophobic protein core seem to restrict large movement of the loop and impede the opening of the hydrophobic pocket for sequestering the acyl chains. NMR studies of the *hydroxybutyryl-* and *octanoyl*-*me*ACP confirm that *me*ACP is unable to sequester the hydrophobic chains in a well-defined central cavity. Instead, *me*ACP seems to interact with the octanoyl tail through a distinct hydrophobic patch without involving large conformational change of loop-2. NMR titration study of the interaction between *me*ACP and the cognate thioesterase partner CalE7 further suggests that their interaction is likely through the binding of CalE7 to the *me*ACP-tethered polyene moiety rather than direct specific protein-protein interaction.

## Introduction

One of the most distinctive features of fatty acid, nonribosomal peptide and polyketide biosynthetic pathways is the utilization of the acyl carrier protein (ACP) or peptidyl carrier protein (PCP) for the shuttling of biosynthetic intermediates among various catalytic domains or proteins [1]–[5]. The small and highly dynamic ACPs or PCPs can be either free-standing proteins or integrated domains within a complex multidomain fatty acid synthase (FAS), polyketide synthase (PKS) or nonribosomal peptide synthase (NRPS). The free-standing ACPs from type II FAS pathways are the best studied ACP systems, with crystallographic and solution NMR studies showing that the type II FAS ACPs adopt a canonical four-helix bundle fold with a binding pocket for sequestering the growing fatty acid chain [6]–[8]. Meanwhile, although the ACP domains from the multidomain type I FASs adopt a similar overall structure, they do not seem to contain a pocket for binding and protecting the growing fatty acid chain [9]–[11]. Recent studies on the integrated ACP domain from the modular type I PKS DEBS and the discrete ACPs from type II PKS have revealed the similarity in overall protein structure but salient differences in the binding of acyl chain between type I and type II PKS ACPs [12]–[17]. Subtle differences between the ACPs in local structure, surface electrostatic potentials and binding mode of biosynthetic intermediates have been also documented. Recent NMR studies further revealed functionally important protein dynamics in PCPs for the modulation of the interaction between the PCPs and the NRPS catalytic domains [5], [18], [19].

CalE8 is an iterative type I PKS that plays a central role in the early stage of the biosynthesis of the naturally occurring enediyne calicheamicin γ_1_^I^ in *Micromonospora echinospora ssp. calichensis*. Distinct from the modular type I PKSs, CalE8 is composed of a single module consisting of several catalytic domains for the synthesis of the putative precursor of the 10-membered enediyne moiety [20]–[24]. The ACP domains of CalE8 and other enediyne PKSs share very low sequence homology with known ACPs [20], [25], [26]. In fact, the initial assignment of the ACP domain in CalE8 was associated with a great degree of uncertainty, not just because of the low sequence homology shared with other ACPs, but also because of the absence of the signature GX(H/D)S(L/I) motif conserved in many ACPs. We previously confirmed the identity of the ACP domain (*me*ACP) in CalE8 through *in vitro* modification of the excised *me*ACP by the phosphopantetheinyl transferase (PPTase) Sfp [27], [28]. Shen and coworkers also demonstrated that the ACP domain of SgcE, a homolog of CalE8 from the biosynthetic pathway of the 9-membered enediyne natural product C-1027, can be phosphopantetheinylated at the predicted Ser site by mass spectrometry [29].

**Figure 1:**
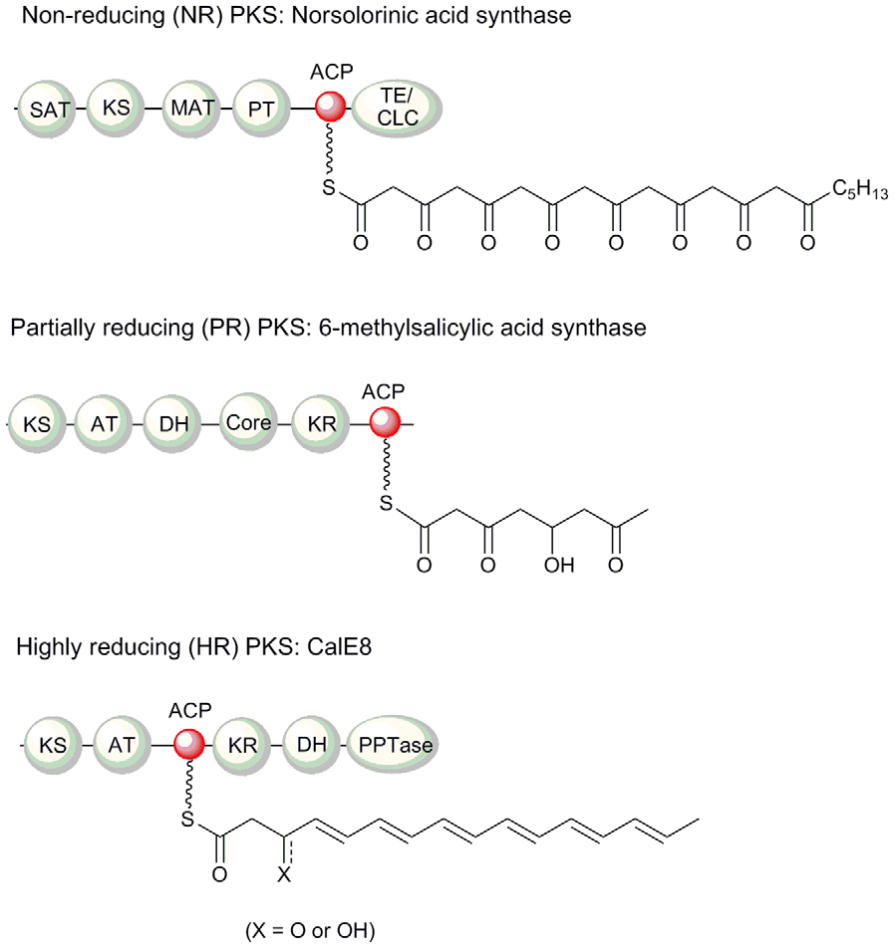
Three classes of type I iterative polyketide synthases (PKS). The proposed PKS products are tethered to the ACP domains.

According to the degree of reduction of *keto* groups in the final polyketide product, iterative type I PKSs have been classified into the non-reducing (NR), partially-reducing (PR) and highly-reducing (HR) families (**Figure 1**) [21], [30]. CalE8 belongs to the HR type I PKS family given that CalE8 and its homologs produce conjugated polyenes under both *in vitro* and *in vivo* conditions [22], [23], [31], [32]. To date, only one structure of the iterative type I PKS ACP domain has been reported [33]. Crump and coworkers determined the solution structure of the ACP domain from the fungal norsolorinic acid synthase (NSAS), which belongs to the iterative NR PKS family [33]. Unlike the polyketide products of the iterative NR and PR PKSs that are cyclized immediately upon formation, the chemically labile conjugated polyenes produced by CalE8 seem to be stabilized by the protein to such an extent that they can be co-purified with the protein [23], [24], [34]. It is thus intriguing to speculate whether the ACP domain plays any role in protecting the conjugated polyenes. Here we report the NMR solution structure of the ACP domain (*me*ACP) of CalE8, which represents the first structure of ACP domains from an HR iterative type I PKS. Studies on the *apo*, *holo* and *acylated me*ACP suggest that the modifications do not alter the overall protein structure but affect the structure at a local level. Two-dimensional NMR spectra of *me*ACP collected in the presence of thioesterase (CalE7) also provide some interesting insight into the transient nature of the interaction between *me*ACP and the cognate protein partner.

## Results

### Overall structure of meACP

**Figure 2:**
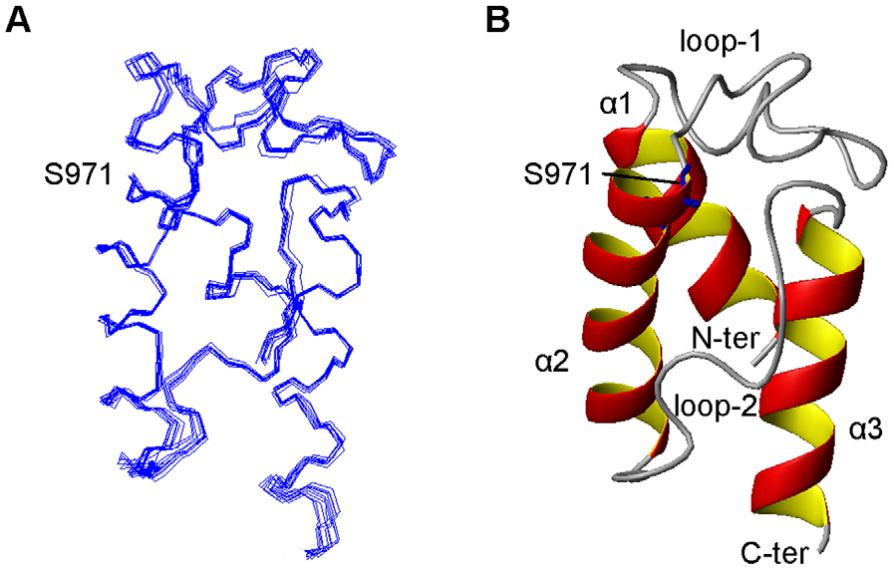
NMR structure of *apo-me*ACP. Residues only from Ala^938^-Glu^1014^ are shown, excluding flexible N- and C- terminal tails. (A) Backbone of an ensemble of the lowest energy conformations shown as line representation (B) Mean *apo-me*ACP solution structure is shown as ribbon model.

The ACP domain construct used in this study encompasses a 92 residue long (Ala^925^-Pro^1016^) fragment of CalE8. Previous studies have confirmed that the *E. coli* overexpressed *me*ACP is in its *apo* form and can be modified by the PPTase Sfp to generate *holo*-*me*ACP [27]. 98% of the residues of the recombinant *apo*-*me*ACP were unambiguously assigned. Unassigned residues are the amino-terminal Met^924^ and Ala^925^. The only disordered segments are the unstructured termini that include Ala^925^ to Thr^936^ and Ala^1015^ to Pro^1016^ (**Figure 2A**). The structured region of *me*ACP has a backbone r.m.s. deviation of 0.44 Å and no distance violation greater than 0.5 Å was observed in our structure calculation (**Table 1**). Ramachandran plot of the final ten structures shows that 98.9% of the residues fall in the allowed region (**Table 1**).

**Table 1:** Experimental restraints and structural statistics for ten lowest-energy NMR structures of *apo-me*ACP out of 100 structures.

NMR distance constraints	
Intra-residue	628
Sequential	324
Medium-range (1<|I-j|<5)	702
Long-range (|I-j|≥5)	654
Total	2308
NMR dihedral restraints	
Φ	62
Ψ	63
Total	125
Structural statistics	
Violations per structure (residues Gly^937^-Arg^1013^)	
NOE violation (Å)	0.41±0.04
Angle violation (°)	3.74±0.39
TALOS >5.0°	0
Max. dihedral angle violation (°)	4.18
Max. distance constraint violation (Å)	0.47
Ramachandran plot region (all residues) [%]	
most favoured	76.3
additionally allowed	19.4
generously allowed	3.2
disallowed	1.1
Mean RMS deviation from the average coordinates (residues Gly^937^-Arg^1013^) (Å)	
Backbone atoms (C^α^, C’, N, O)	0.44±0.12
All heavy atoms	1.10±0.11

Overall, *me*ACP assumes a globular fold of a twisted three-helix bundle (*α*1 [Ala^938^-Ala^950^], *α*2 [Ser^971^-Met^984^] and *α*3 [Val^1001^-Glu^1014^]) and two well-defined long loops (loop-1 [Glu^951^-Ser^970^] and loop-2 [Met^985^-Thr^1000^]) (**Figure 2B**). The domain seems to be stabilized through the packing of the hydrophobic side chains of the residues originated from the three helices as well as the two loops. The estimated helical content of 44.6% based on 41 out of 92 residues, is significantly lower than that of other known ACPs, which usually have more than 50% of helical content [35]. Most characterized ACPs adopts a canonical four-helix bundle structure with helix *α*1 running almost anti-parallel to helices *α*2 and *α*4, as well as a short helix *α*3 within the loop-2 that connects the helices *α*2 and *α*4. In *me*ACP, the region where the short helix should be located seems to be disordered and the lack of the local helical feature contributes to the low helical content of the protein. Some ACPs and PCPs have been reported to adopt two or more inter-converting conformations [18], [36]. But we did not observe a significant sub-population for *me*ACP.

### Local structural features

**Figure 3:**
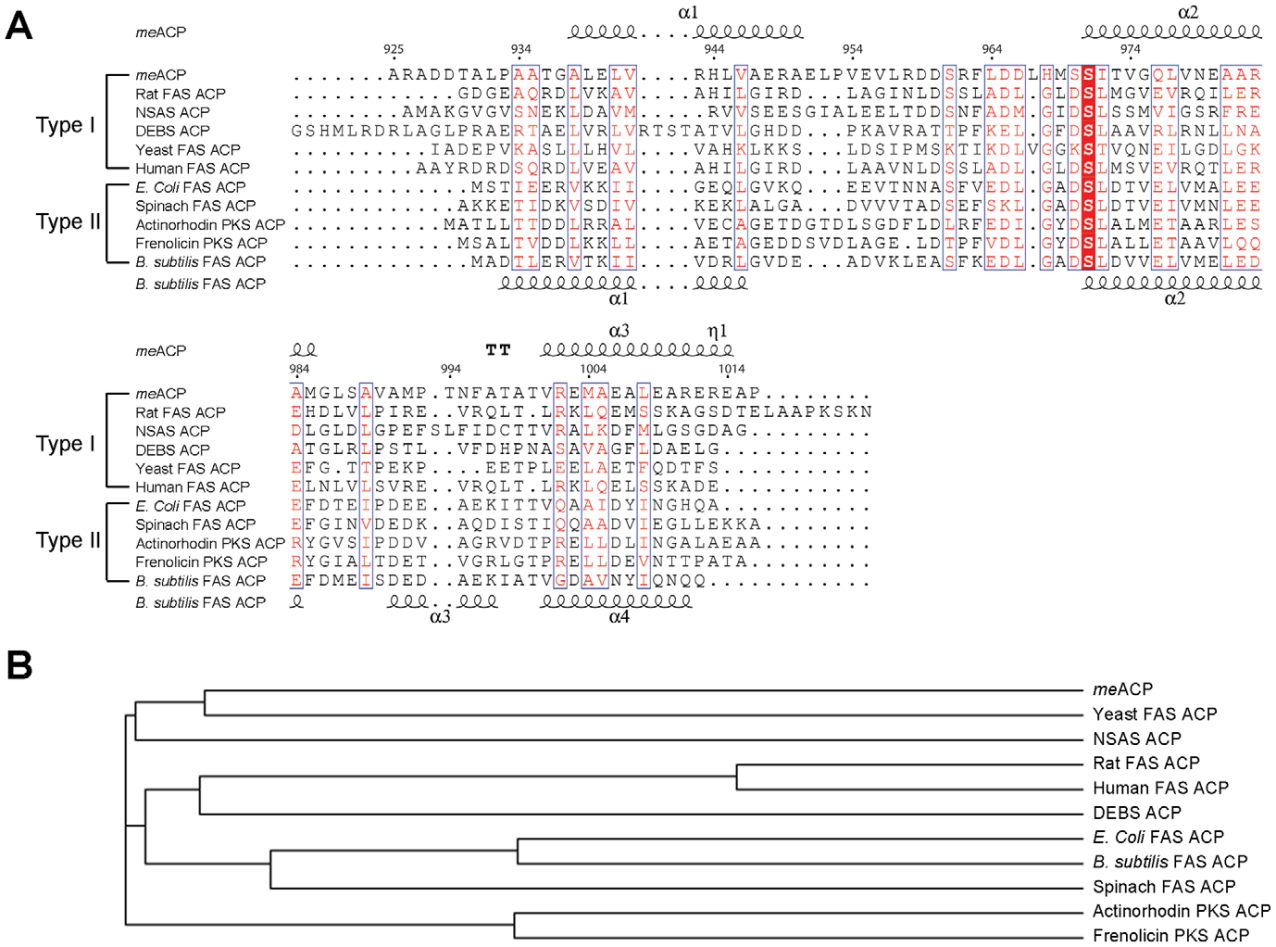
Comparison of *me*ACP with other ACPs. (A) Multiple sequence alignment of *me*ACP with selected type I and type II ACPs. The secondary structures of *me*ACP (top) and *B. subtilis* FAS ACP (bottom) are shown. The phosphopantetheine attaching serine site is shaded in red. (B) Phylogenetic analysis of the type I and II ACPs from (A).

**Figure 4:**
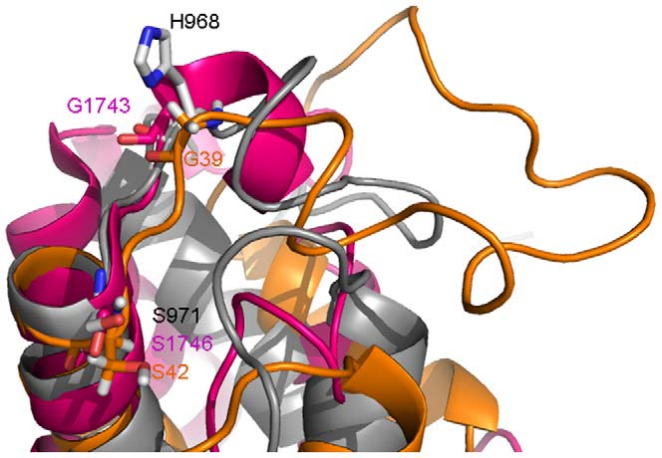
Conformation of the motif harboring the phosphopantetheine attaching serine in *me*ACP and two other ACPs. The GX(H/D)S(L/I) motif in type I NSAS PKS ACP (PDB ID: **2KR5**) (pink) and type II actinorhodin PKS ACP (PDB ID: **1AF8**) (orange) and the HMSSI motif in *me*ACP (grey) are shown as ribbons. The conserved Gly and Ser residues in **G**X(H/D)**S**(L/I) motif are shown as stick. The His and Ser residues of the **H**MS**S**I motif in *me*ACP are also shown as stick.

A comparison with other ACP structures reveals some salient features of *me*ACP. Despite the facts that *me*ACP shares very low sequence homology with other ACPs and it lacks the signature GX(H/D)S(L/I) motif conserved in many ACPs (**Figure 3A**), the putative phosphopantetheine attachment site (Ser^971^) is still located at the beginning of helix *α*2 **(** **Figure 4** **)**. The orthodox GX(H/D)**S**(L/I) motif is replaced by a H^968^MS**S** ^971^I motif in *me*ACP with His^968^ and Ser^970^ substituting the canonical Gly and Asp/His residues. The essential role of Ser^971^ was confirmed by the observation that S971A mutation completely abolished the activity of CalE8 (data not shown). The HMS^970^ triad constitutes part of loop-1 with the solvent-exposed side chains His^968^, instead of the typical Gly residue conserved among many characterized ACPs (**Figure 4**). In addition, Ser^970^ replaces an Asp residue commonly found in other type I and II ACPs. This Asp from *B. subtilis* FAS ACP forms a salt bridge with the Arg^14^ of ACP synthase (ACPS) [37]. Thus, considering that such residues preceding Ser^971^ have been known to affect protein-protein interaction in some ACPs and given the different physio-chemical property of the residues, a dissimilar protein surface of *me*ACP may be involved in the recognition of some of its partners.

One of the common features of types II FAS or PKS ACPs is that they contain a helix *α*2 that is rich in acidic residues. The clustered negative charges on helix *α*2 are considered to be critical for the recognition of the free-standing ACPs by their protein partners. Helix *α*2 is therefore known as the universal ‘recognition helix’ in carrier proteins. Several acidic residues in helix *α2* of type II ACPs can form salt bridges with positively charged residues on their protein partners [38]. However in type I FAS ACP domains the helix *α*2 seems to be less negatively charged, presumably because the specific domain-domain interaction driven by charged-charged interactions is less critical in the multidomain type I FAS system. In accordance with other type I ACPs, the helix *α*2 of *me*ACP only contains a single acidic residue (Glu^980^). The resemblance of *me*ACP to type I ACPs is also consistent with the phylogenetic relationship of *me*ACP with other carrier proteins (**Figure 3B**). In contrast to the relatively neutral helix *α*2, the adjacent loop-1 harbors a large number of highly charged residues including Glu^951^, Glu^955^, Arg^958^, Asp^959^, Asp^960^, Arg^962^, Asp^965^, Asp^966^ and His^968^. The enrichment of the charged residues on loop-1 is not uncommon and has also been seen in some ACPs, such as the ones from the biosynthetic pathways of frenolicin and norsolorinic acid [33], [39]. Given the proximity of loop-1 near the recognition helix *α*2, these charged residues might provide the specific docking interface for other protein domains or stabilize the phosphopantetheine arm.

**Figure 5:**
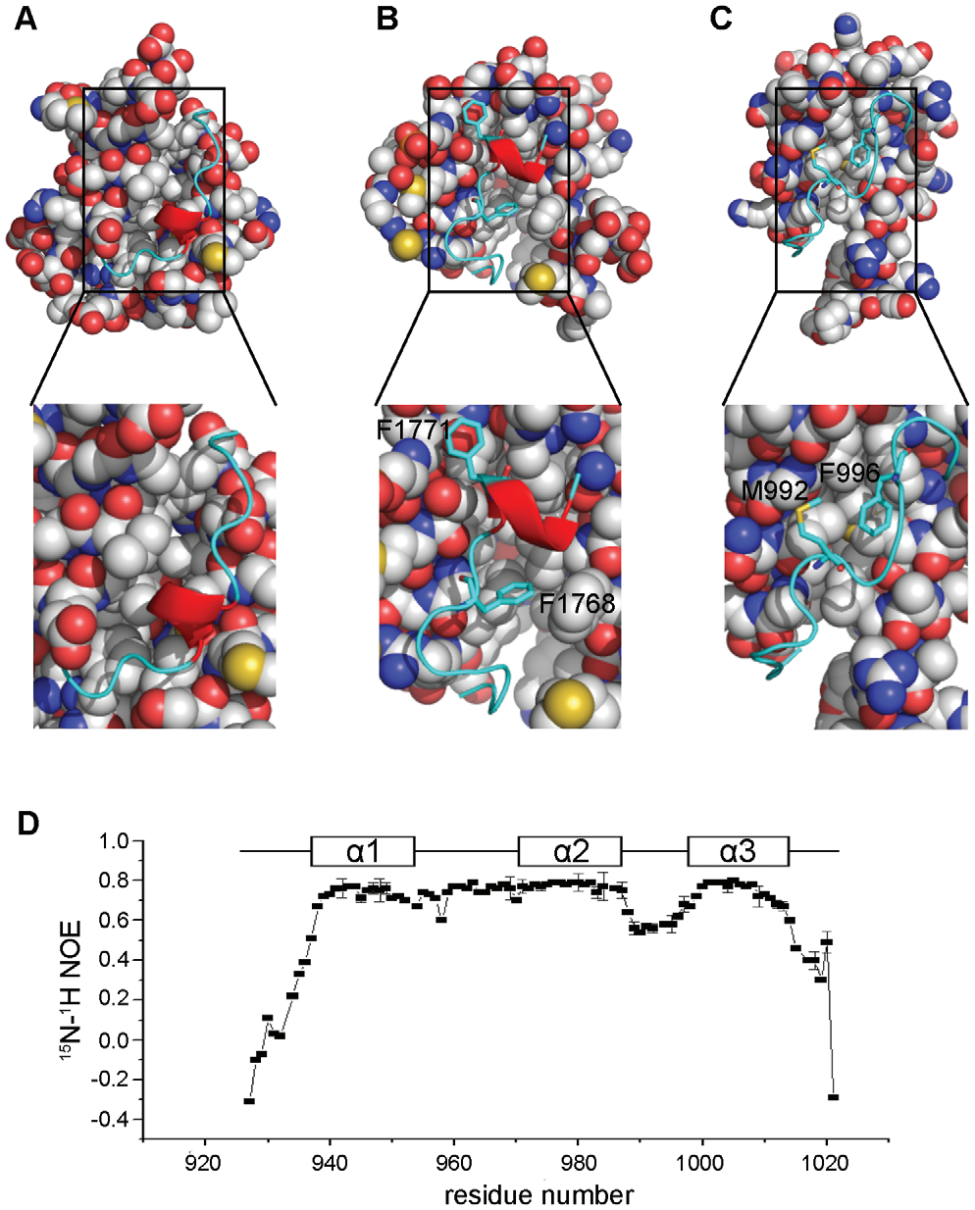
Conformation mobility of loop-2. (A) Type II actinorhodin PKS ACP (PDB ID: **1AF8**) that contain mainly small residues in loop-2. (B) Type I NSAS PKS ACP (PDB ID: **2KR5**) with the two bulky residues (Phe^1768^ and Phe^1771^) highlighted (C) *me*ACP with the two bulky residues (Phe^996^ and Met^992^) packed against the hydrophobic pocket highlighted. (D). ^1^H-^15^N NOE plot of *apo-me*ACP acquired at an 800 MHz spectrometer at 25°C. The positions of the helices are indicated.

It has been suggested that loop-2, which contains a short helix for some ACPs, serves as a conformational switch in sequestering ACP domain-tethered acyl chains in type II FAS systems [5]. With the exceptions of *act* ACP and *B. subtilis* FAS ACP [6], [16], all the characterized ACPs contain a short helix within loop-2. The short helix seems to be absent in *me*ACP as well. In addition, in the case of type II ACPs, loop-2 is largely comprised of charged or small non-polar residues that allow the opening of a hydrophobic cleft for binding acyl intermediates (**Figure 5A**); whereas loop-2 of type I ACPs, such as NSAS ACP, usually contains bulky residues that are packed against the protein core to prevent the opening of the binding cleft (**Figure 5B**) [33]. In *me*ACP, loop-2 also contains two hydrophobic residues (Met^992^, Phe^996^) with their bulky side chains packed against the hydrophobic core of the protein (**Figure 5C**). Although the packing of Met^992^ and Phe^996^ may restrict the conformational flexibility of loop-2, ^1^H-^15^N NOE plot of *me*ACP indicates that the residues of loop-2 (Gly^986^-Thr^1000^) are still significantly more flexible than the residues of loop-1 and helices *α*1, 2 and 3 (**Figure 5D**). The observed conformational flexibly of loop-2 raises the tantalizing possibility that it may undergo a large conformational change to open a protein pocket for binding the PKS products.

### Comparison between apo- and holo-meACP

**Figure 6:**
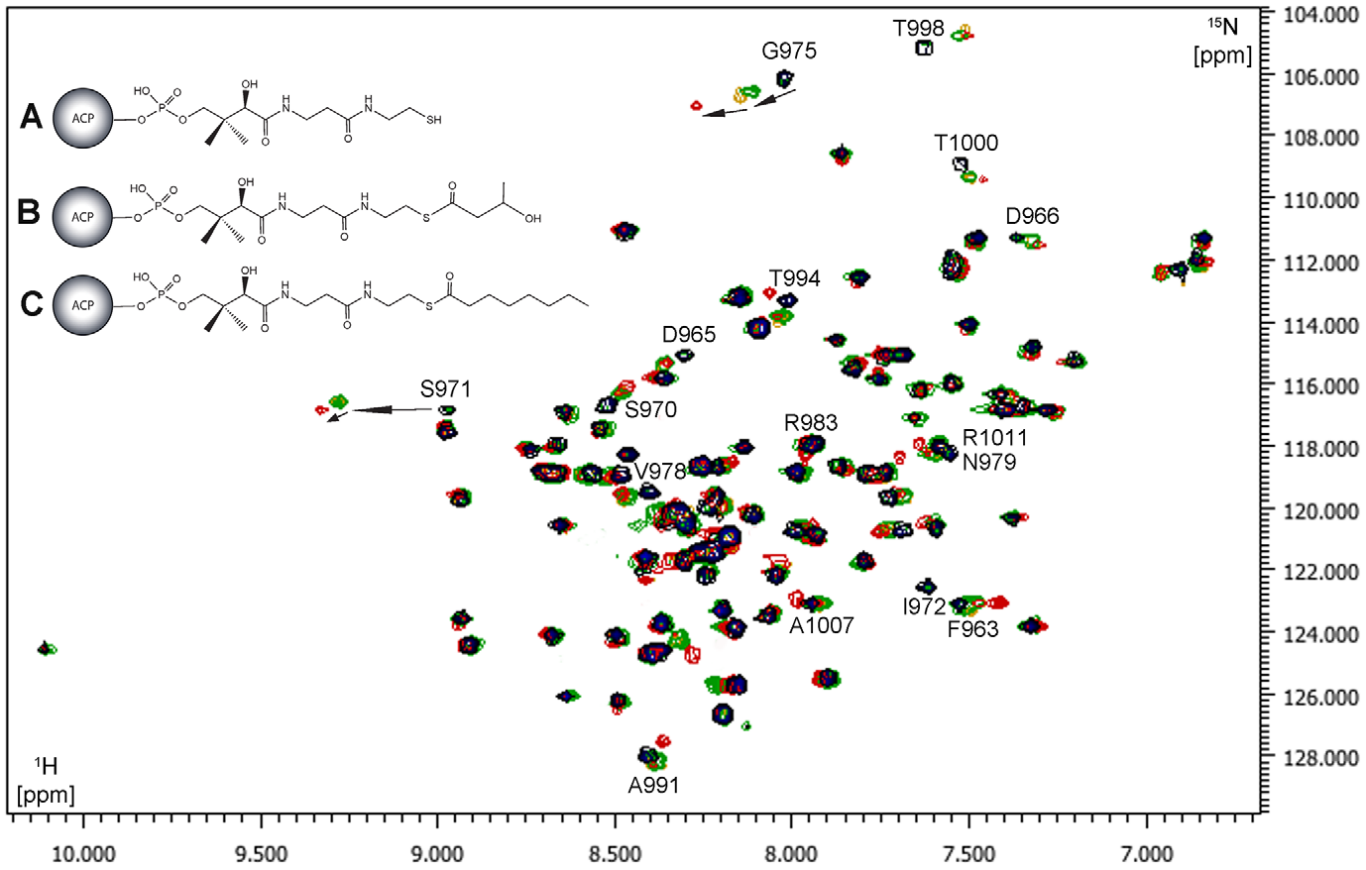
Overlaid ^1^H-^15^N HSQC spectra of *apo-me*ACP and its derivatives. *apo*-*me*ACP (black), *holo-me*ACP (A, yellow), *hydroxybutyryl-me*ACP (B, green) and *octanoyl-me*ACP (C, red) acquired at 15°C. The change of chemical shifts for the residues Ser^971^ and Gly^975^ from *apo*, *holo*, *hydroxybutyryl* to *octanoyl-meACP* are indicated by the black arrows.

**Figure 7:**
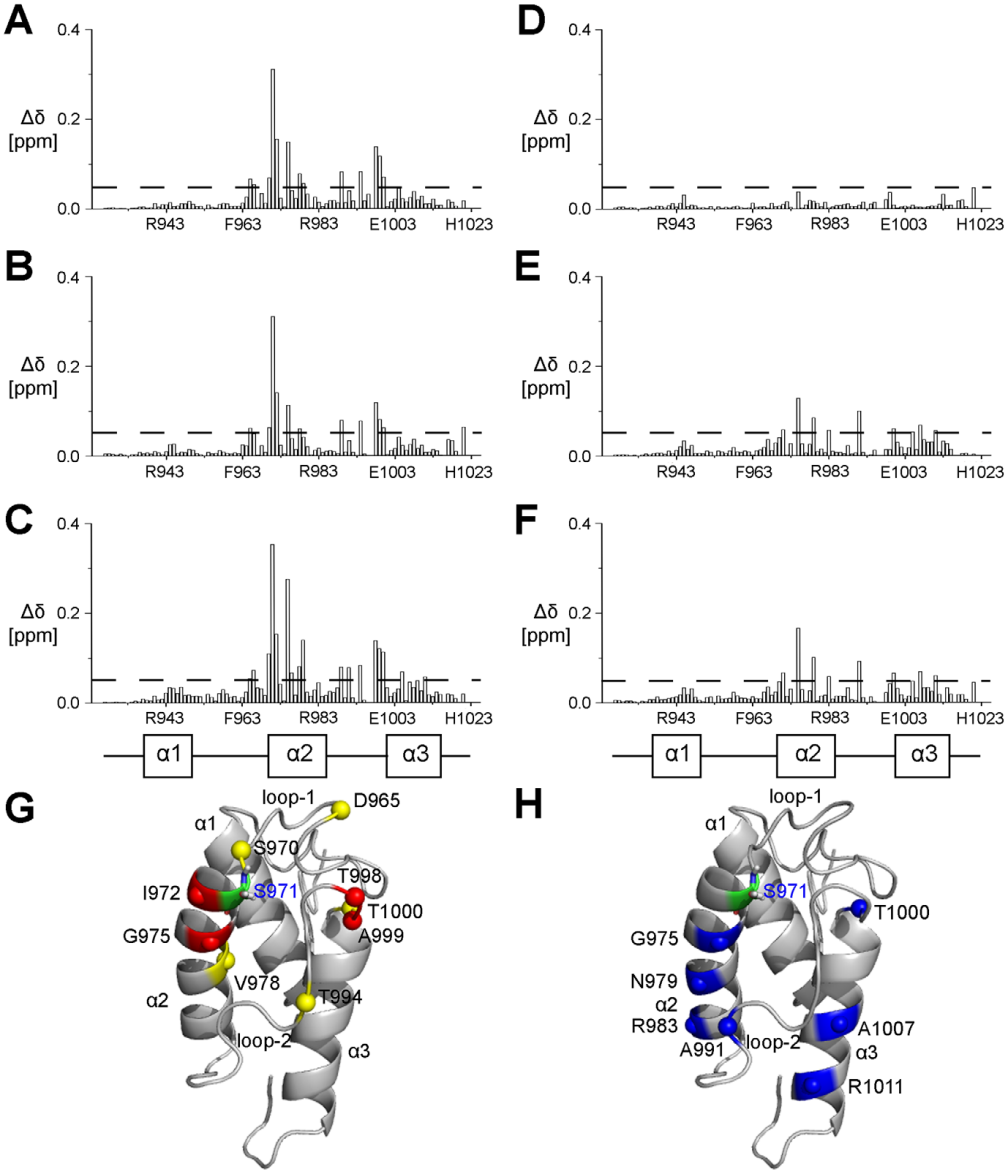
Chemical shift perturbations (Δδ) of *me*ACP caused by phosphopantetheinylation and acylation. Combined chemical shift change plots of *apo*-*me*ACP versus (A) *holo*-*me*ACP, (B) *hydroxybutyryl*-*me*ACP and (C) *octanoyl-me*ACP. Combined chemical shift change plots of (D) *hydroxybutyryl-me*ACP versus *holo-me*ACP, (E) *octanoyl-me*ACP versus *holo*-*me*ACP and (F) *octanoyl-me*ACP versus *hydroxybutyryl-me*ACP. A combined chemical shift cut-off of 0.05 ppm is shown as the dashed line. The residue number and the positions of the helices (*α*1, *α*2 and *α*3) are also indicated. (G) Ribbon representation of *apo-me*ACP with the positions of the perturbed residues upon phosphopantetheinylation are highlighed (combined chemical shift cut-off >0.05 ppm (yellow sphere) and >0.10 ppm (red sphere)). (H) Ribbon representation of *apo-me*ACP with the positions of the perturbed residues based on (F) upon acylation by octanoyl moiety are highlighted (combined chemical shift cut-off >0.05 ppm (blue sphere)).

ACP domain only becomes fully functional after conversion to *holo* protein with a phosphopantetheine group attached to the invariant Ser residue. To find out whether the presence of the phosphopantetheine group perturbs the *me*ACP structure and dynamics, the *holo* form of the protein was prepared by incubating *me*ACP with coenzyme A and the PPTase Sfp. Comparing the HSQC spectra of *apo*- and *holo*-*me*ACP, we found that the overall structures of the two forms are essentially the same (**Figure 6**). Nonetheless, Ser^971^ exhibits a large shift from the *apo* form (^1^H: 8.97 ppm, ^15^N: 116.80 ppm) to the *holo* form (^1^H: 9.28 ppm, ^15^N: 116.50 ppm) or a combined chemical shift change of 0.31 ppm. The presence of the phosphopantetheine group also clearly affects backbone amides of the neighboring residues that include Asp^965^ and Ser^970^ from loop-1, Ile^972^, Gly^975^ and Val^978^ from helix *α*2 and Thr^994^, Thr^998^, Ala^999^ and Thr^1000^ from loop-2 (**Figure 7A and 7G**). These perturbed residues are mostly located at protein surface in the vicinity of Ser^971^ (with the exception of Val^978^ that is probably due to a subtle conformational change in helix *α*2). The observation indicates that the *me*ACP-attached phosphopantetheinyl group is most likely to be exposed to solvent and accessible to other catalytic domains.

### Interaction of acylated moiety with meACP

One of the most interesting findings about PKS/FAS and NRPS is the presence of a ligand binding pocket on the surface of type II ACPs or PCPs for the binding of the growing intermediates. For example, the sequestering of aliphatic segment of decanoate (10:0) or stearate (18:0) in the binding pocket of spinach ACP induces striking perturbations of HSQC peaks [40], [41]. On the other hand, hexanoyl (6:0) or palmitoyl (16:0) chain attached to type I rat FAS ACP showed little changes of HSQC peaks, suggesting that the rat FAS ACP does not bind the aliphatic chain [9].

Given the observed conformational flexibility of loop-2 in *me*ACP, we asked whether type I *me*ACP can also bind the chemically labile polyene intermediates in the same hydrophobic pocket that accommodates the side chains of Met^992^ and Phe^996^. Loading *me*ACP with the highly conjugated polyenes is impractical given the chemical reactivity of the polyenes. Instead, we used hydroxybutyryl- and octanoyl- groups to mimic the aliphatic chains of the polyene products. Overall, only minor perturbations in ^1^H-^15^N HSQC spectrum were observed between the *holo-* and *acylated-me*ACPs, suggesting that residues lining the putative binding pocket are not disturbed (**Figure 6**). However, although only negligible differences between the *holo*- and *hydroxybutyryl-me*ACP (combined chemical shift differences <0.05 ppm) were observed, extending the acyl chain from hydroxybutyryl to octanoyl causes greater and more extensive chemical shift changes (0.05–0.20 ppm) (**Figure 7B-F**). With a chemical shift change cutoff of 0.05 ppm, eight residues that include Ser^971^, Gly^975^, Asn^979^, Arg^983^, Ala^991^, Thr^1000^, Ala^1007^ and Arg^1011^ exhibit greater perturbation in *octanoyl-me*ACP than *hydroxybutyryl-me*ACP. Note that the three residues Ser^971^, Gly^975^ and Thr^1000^ are affected by both phosphopantetheinylation and extension of the acyl chain, indicating that elongation of acyl chain may also alter the position or conformation of the phosphopantetheinyl moiety. These perturbed residues of *octanoyl-m*eACP are mapped on the protein and most of them are located on helix *α*2, loop-2 and as well as helix *α*3 (**Figure 7H**).

### Interaction between meACP and the thioesterase CalE7

*me*ACP domain must interact with the ketosynthase (KS), acyltransferase (AT), Ketoreductase (KR), dehydratase (DH) and PPTase domains of CalE8 for the synthesis of the polyene product as well as the thioesterase CalE7 for hydrolytic release of the product. It would be interesting to know the molecular basis of the interaction between *me*ACP and the catalytic domain of CalE8 as well as CalE7. However, cloning and expression of the stand-alone catalytic domains of CalE8 failed to produce soluble proteins. We were only able to examine the interaction between *me*ACP and the hot-dog fold thioesterase CalE7 in an attempt to map out the recognition surface of *me*ACP for CalE7. When we first determined the crystal structure of CalE7, a few charged residues at the entrance of the substrate-binding tunnel were speculated to be involved in binding *me*ACP [34]. However, when the thioesterase CalE7 was titrated into the ^15^N-labeled *apo*- and *holo*-*me*ACP protein solution for NMR measurement, no HSQC peak was perturbed in both chemical shifts and intensity even when the ratio of CalE7 to *me*ACP reached 2.5∶1. This indicates that the interaction between CalE7 and *me*ACP is too transient or weak to be detected (**Figure S1 and S2**). The transient nature of the CalE7 and *me*ACP interaction is further supported by the observation that none of the charged residues at the substrate-binding channel is conserved in the homologous DynE7 protein [42]. In conjunction with the observation that a catalytic incompetent mutant of CalE7 (but not the wild type CalE7) can form stable 1∶1 complex with CalE8 with the conjugated polyene products remaining attached to the ACP domain [42], it is reasonable to believe that the interaction between *me*ACP and CalE7 is rather weak and the recognition is through the binding of the nascent polyene product in the hydrophobic substrate channel of CalE7. The weak transient interaction will be further explored using paramagnetic relaxation enhancement approaches.

## Discussion

As the first structure determined for the ACP domain of a highly-reducing (HR) iterative type I PKS, *me*ACP shares extremely low sequence homology with other known ACPs, including the ACP domain of the non-reducing (NR) iterative type I PKS NSAS (12% identity). *me*ACP also features an unusual HMS**S**I motif (or H(L/M)(S/T/N)**S**(I/L) for *me*ACP homologs) harboring the putative Ser ^971^ phosphopantetheinylation site, in contrast to the more common GX(H/D)**S**(L/I) motif seen in other ACPs. We have previously performed site-directed mutagenesis to show that Ser^971^, but not Ser^970^, is required for the enzymatic function of CalE8 (data not shown). The specificity towards Ser^971^ must be determined by the location of the residue and its proximity to the CoA substrate in the *me*ACP-PPTase complex. As there is a ∼7.0 Å distance between the two hydroxyl groups for the two Serine residues, it is unlikely for the PPTase domain to modify the wrong Serine (Ser^970^).

Despite the low sequence homology and the uncommon HMSSI motif, *me*ACP adopts a helix-bundle structure that is highly similar to other ACPs with the exception of the absence of the short helix within loop-2. As a result of the missing short helix, the structure of *me*ACP is best described as a twisted three-helix bundle instead of the canonical four helix-bundle structure. In addition, the relatively shortage of negative charges on the ‘recognition helix’ (*α*2) of *me*ACP is consistent with the observations for other type I FAS and PKS ACPs, presumably resulted from the lack of evolutionary selection pressure on type I ACP domains for specific domain-domain interactions within the multidomain FAS or PKS protein.

It is well known that loop-2 of type II FAS and PKS ACPs generally consists of charged and less bulky residues to confer great conformational mobility to the loop; whereas type I ACPs tend to contain bulky hydrophobic residues on the loop to restrict the mobility of the loop through hydrophobic interaction with the protein core. In light of this, *me*ACP harbors two non-polar residues (Met^992^ and Phe^996^) with the side chains packed against the protein core. Similar hydrophobic interactions were recently reported for the ACP domains of the rat type I FAS, the non-reducing (NR) types I iterative PKS NSAS and the type I modular PKS DEBS [9], [33]. The protection of the intermediates is considered to be particularly important for type II FASs and PKSs because the free-standing ACPs need to transport the intermediates from one protein to another in the cytoplasm. The conformational mobility of loop-2 has been suggested to be crucial for the opening of a hydrophobic cleft for sequestering and protecting acyl intermediates. Such protection, however, is less critical for type I FASs and PKSs with the integrated ACP domain and intermediates probably already shielded from solvent by the surrounding catalytic domains. The structural observations thus seem to reinforce the view that restricted conformational mobility of loop-2 resulted from interaction of the non-polar residues may be a common feature for the ACP domains of the multidomain type I FAS and type I iterative PKS.

**Figure 8:**
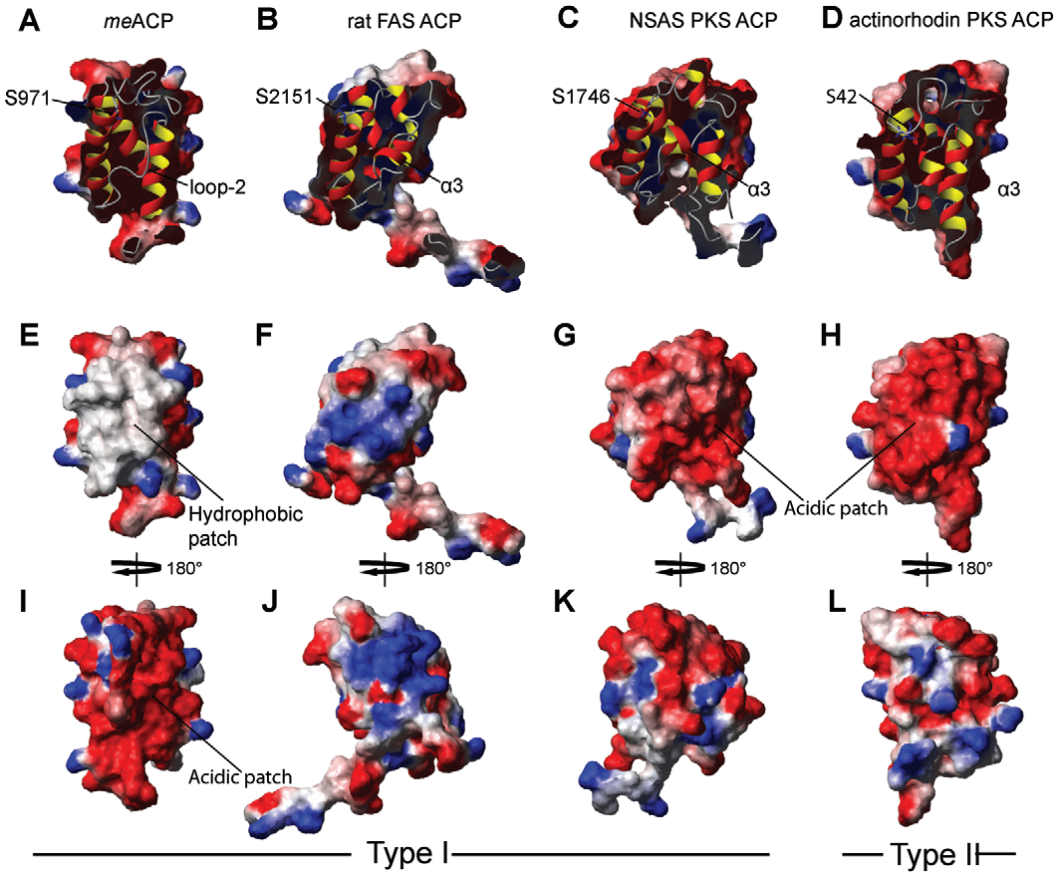
Embedded ribbon representation and electrostatic potential surface of ACPs. Type I *me*ACP (A, E and I), rat FAS ACP (B, F and J, PDB ID: **2PNG**), NSAS PKS ACP (C, G and K, PDB ID: **2KR5**) and Type II actinorhodin PKS ACP (D, H and L, PDB ID: **1AF8**). The invariant Ser is shown as blue stick and labeled. The protein surfaces are colored as white (hydrophobic), blue (basic) and red (acidic) with the same electrostatic potential scale.

**Figure 9:**
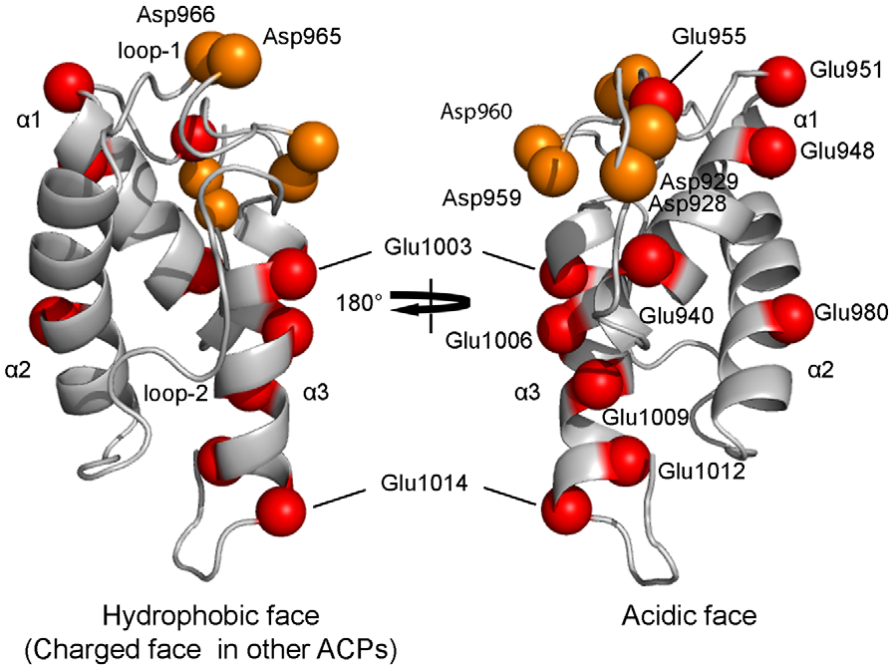
Ribbon representation of *apo-me*ACP with either hydrophobic or acidic face. The hydrophobic and Glu-Asp acidic faces are shown with highlighted spheres colored red (Glu) and orange (Asp).

Despite the hydrophobic interaction between Met^992^ and Phe^996^ with the protein core, the ^1^H-^15^N NOE plot revealed relatively greater conformational flexibility for loop-2 than loop-1 and the three helices (**Figure 5D**). This observation first raised the question whether loop-2 of *me*ACP still can undergo conformation change to create a transient protein pocket for binding the highly hydrophobic polyene products. Attachment of 4′-phosphospanteine group and the additional hydroxybutyryl group to the Ser^971^ site has little effect on the overall protein fold, despite minor local perturbations restricted to residues in the vicinity of Ser^971^ (**Figure 7**). These observations suggest that the phosphopantetheine and hydroxybutyryl moieties are unlikely to be bound in a protein pocket and thus remain in the bulk solvent. In contrast, the NMR spectra of the *octanoyl-me*ACP show more extensive perturbations, with the perturbed residues distributed in a region flanked by helix *α*2, loop-2 and helix *α*3 (**Figure 7H**). The magnitude of the perturbation is considered to be relatively small compared to that for the type II ACPs that sequester the acyl chain through a significant conformational change [13], [43], Hence, the results do not seem to support that *me*ACP is able to undergo dramatic conformational change and sequester the PKS product in a well-defined protein pocket. Instead, examination of the electrostatic potential surface of *me*ACP reveals two distinct patches, a unique hydrophobic patch and an acidic patch unlike other ACPs currently deposited in Protein Data Bank (PDB) (**Figure 8**). The rather common charged patch encompassing the recognition helix *α*2 observed in other ACPs, is replaced by an unusual hydrophobic patch in *me*ACP (**Figure 8E**). *me*ACP also features an acidic patch that consists mainly Glu residues, including Glu^940^ and Glu^948^ from helix *α*1, Glu^951^ and Glu^955^ from loop-1, Glu^980^ from helix *α*2, and Glu^1003^, Glu^1006^, Glu^1009^, Glu^1012^ and Glu^1014^ from helix *α*3. (**Figure 8I** **and** **Figure 9**). No such hydrophobic or acidic patch has been observed in other type I ACPs, such as the rat FAS ACP and NSAS PKS ACP or type II ACPs such as actinorhodin PKS ACP (**Figure 8**) [33]. Given the location of the hydrophilic patch and the observed NMR perturbations with the eight residues in the distinct hydrophobic surface of *me*ACP, it is feasible that the non-polar octanoyl chain may interact with hydrophobic patch to avoid the unfavorable solvation of the hydrophobic groups. Such hydrophobic interaction may play a significant role in the stabilization of unsaturated polyene intermediates during chain elongation. Alternatively, the hydrophobic patch could be conserved for domain-domain interaction. The latter remains a possibility considering that this region also encompasses the helix *α*2 that is most likely to be involved in protein-protein interaction.

The biosynthetic mechanisms for the 10- and 9-membered enediyne moieties and the precise function of CalE8 and its homologs are currently under intensive investigation. The first structure of the HR iterative PKS ACP domain presented here not only reveals some interesting structure features that reinforce some of the views held for type I FAS and PKS ACPs, but also discloses some unique characteristics of the *me*ACP domain. The lack of a strong negatively charged helix *α*2 and the moderate conformational flexibility of loop-2 are consistent with the role of the *me*ACP domain in shuttling acyl intermediates within the catalytic chamber of the multidomain PKS CalE8. The absence of the short helix within loop-2 is rare but not totally unprecedented. The interaction between the acyl aliphatic chain and the hydrophobic patch is distinct from the well-established interaction between acyl chains and the well-defined protein pocket. Although the biological significance of the hydrophobic patch and highly acidic patch remain to be established, the hydrophobic patch may be involved in the interaction with other catalytic domain, or play a role by interacting and stabilizing the intermediates and products of CalE8. Lastly, the lack of significant binding between *me*ACP and its cognate protein partner CalE7 indicates that specific strong protein-protein recognition is not crucial for CalE7 to bind and perform the hydrolytic cleavage on the product of CalE8.

## Materials and Methods

### Protein cloning, expression and purification

The cloning of *me*ACP domain has also been described previously [27]. The ACP and catalytic domains of CalE8 were cloned into compatible vectors and transformed into BL21 cells for expression. Seed culture of BL21 cells containing the respective plasmids were grown overnight before being inoculated into large scale cultures. The cultures were allowed to grow at 37°C and 200 rpm until the OD_600nm_ reached 0.6. Induction was done with IPTG at 0.4 mM concentration. Cells were harvested 20 hours later after being left to grow at 16°C and 160 rpm. The cell pellet was then resuspended in lysis buffer (50 mM HEPES at pH 7.5, 300 mM NaCl, 5% glycerol, 5 mM β-mercaptoethanol). Subsequently the cells were lysed by sonication. The supernatant obtained after high speed centrifugation was applied onto Ni^2+^-NTA column for purification with a stepped gradient. The final eluent containing the recombinant protein was applied onto gel-filtration column Superdex75 or Superdex200 depending on the size of the individual protein for further purification in a buffer suitable for NMR experiments (50 mM NaH_2_PO_4_/Na_2_HPO_4_ at pH 6.85, 50 mM NaCl, 1 mM DTT). The fractions containing the protein were pooled and concentrated. After that, the proteins solutions were stowed away in the −80°C freezer for future NMR analysis. The same expression and purification protocols were used to prepare ^15^N, ^13^C-labeled *me*ACP protein by using an M9 medium containing ^15^N-isotopic ammonium chloride and ^13^C isotopic glucose. The labeled protein obtained from gel filtration was further purified with a Mono Q column. A refined gradient was employed using buffer A (50 mM NaH_2_PO_4_/Na_2_HPO_4_, 1 mM DTT) and buffer B (50 mM NaH_2_PO_4_/Na_2_HPO_4_, 1 M NaCl, 1 mM DTT). The salt gradient increased gradually from 0% to 40% of B in 50 ml. The majority of the *me*ACP did not bind to the column and was eluted at early stage of the gradient. The fractions containing *me*ACP were pooled and concentrated for NMR as well as acylation experiments. The thioesterase CalE7 were cloned, expressed and purified as described [23], [34]. The PPTase Sfp used for modification of *me*ACP was prepared according to the established procedure [27].

### Preparation of holo- and acylated-meACP

The reaction for the production of *holo-* and *hydroxybutyryl-me*ACPs were summarized as follows: 400 µl of ACP (12 mg/ml), 100 µl of Sfp (68 mg/ml), 40 µl of CoA or acyl-CoA (50 mM) and 10 µl of MgCl_2_ (1 M) were mixed together in 1 ml of reaction buffer (100 mM Tris at pH 8.2, 300 mM NaCl, 1 mM DTT). The reaction was carried out at 30°C for 8–12 hours. The progress of the conversion was monitored by HPLC analysis using an eclipse XDB RP C8 column at 2 hour intervals. The gradient employed was from 10% of acetonitrile to 90% acetonitrile in half an hour. Elution of *me*ACP and Sfp were monitored at the wavelength of 220 nm. Upon completion of the reaction, the mixture was desalted with NaH_2_PO_4_/Na_2_HPO_4_ buffer without NaCl to bring down the NaCl concentration to ∼50 mM. Aforementioned refined gradient was employed using MonoQ for the separation of *holo-* or *acylated-me*ACP from Sfp. A gel filtration step with Superdex-75 column was added to further improve the purity of the *holo-* and *hydroxybutyryl-me*ACP. For the production of *octanoyl-me*ACP, the reaction buffer was modified to have the following composition: 50 mM NaH_2_PO_4_/Na_2_HPO_4_ at pH 7.0, 200 mM NaCl, and 1 mM DTT. The duration of the reaction was prolonged to 16 to 20 hours at 25°C and the conversion rate for the modifications is >95%.

### NMR spectroscopy

All NMR experiments were performed on an 800 MHz NMR spectrometer (Bruker) unless otherwise indicated. All NMR samples including ACPs and CalE7 were prepared in a buffer with 50 mM NaH_2_PO_4_/Na_2_HPO_4_ at pH 6.85, 50 mM NaCl, 1 mM EDTA, 1 mM DTT, 5% D_2_O. Because the *me*ACP protein has weak UV absorbance at 280 nm due to the lack of Trp residues, the protein concentrations were determined by the Bradford assay at 595 nm. To determine the structure of *apo*-*me*ACP, we collected the following NMR spectra on a ∼1 mM ^13^C,^15^N-labeled protein sample: 2D ^1^H-^15^N HSQC, 2D ^1^H-^13^C HSQC, 3D HN(CO)CA, 3D HNCA, 3D MQ-(H)CCH-TOCSY[44] and 4D timeshared ^13^C/^15^N edited-NOESY [45]. To measure the heteronuclear NOEs, two spectra without and with proton saturation were measured on a ∼1 mM ^15^N-labeled sample with a saturation delay of 4 s and recycle delay of 4 s by the inverse-detected 2D NMR method. Proton saturation was achieved by a train of 120° pulses spaced at 5 ms.

In the thioesterase CalE7 titration experiments, an initial concentrated unlabeled CalE7 protein solution (0.8 mM) was added to ^15^N-labeled *apo-me*ACP (0.3 mM) or ^15^N-labeled *holo-me*ACP (0.3 mM). The samples were first allowed to incubate for 10 min prior to NMR data collection. TROSY-HSQCs were recorded at three CalE7 concentrations (0 mM, 0.22 mM and 0.38 mM) in the same ACP buffer till a final [*me*ACP] of 0.15 mM or molar ratio [CalE7]: [*me*ACP] of 2.5∶1. All data were processed with NMRpipe and analyzed with NMRspy and an extension XYZ4D (http://yangdw.science.nus.edu.sg/Software&Scripts/XYZ4D/index.htm). NMRspy was recently developed to facilitate resonance assignment with the 4D NOESY-based strategy [46].

^1^H-^15^N HSQCs of *apo-*, *holo-*, *hydroxybutyryl-* and *octanoyl-me*ACP (0.1 mM) were acquired at 15°C instead of 25°C on an 800 MHz NMR spectrometer. This allows us to observe Ser^971^ which was predominantly weak at higher temperature >20°C using 0.1 mM of acylated ACPs. The combined chemical shift perturbation was calculated using equation 1:
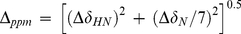
(1)where Δδ_HN_ and Δδ_N_ are the respective differences of ^1^H and ^15^N chemical shifts between different forms of *me*ACP: *apo-me*ACP, *holo-me*ACP or *acylated-me*ACP (*hydroxybutyryl-* and *octanoyl-me*ACP).

### Structure calculation

NOE restraints were obtained from the timeshared 4D ^13^C/^15^N-edited NOESY (containing ^13^C, ^15^N-edited, ^13^C, ^13^C-edited and ^15^N, ^15^N-edited subspectra) using NMRspy. -Backbone torsional angle restraints, Φ and Ψ were obtained from chemical shift data using TALOS [47]. Ambiguous NOEs were assigned with the iterative structure calculation method using Cyana 2.1 [48]. The final 10 lowest energy structures out of 100 calculated were selected. The quality of the structure was assessed using PROCHECK [49].

The NMR assignment data is deposited in the BioMagResBank under BMRB accession number 17355. The coordinates of the ensemble of 10 structures have been deposited in the Protein Data Bank (PDB ID: 2L9F).

## Supporting Information

Figure S1 **^1^H-^15^N HSQC of ^15^N-labeled** ***apo-me*** **ACP titrated against unlabeled CalE7.** The titration is performed at 25°C in monomeric *apo-me*ACP: CalE7 molar ratio of (A) 1∶0 (black) (B) 1∶1 (blue) (C) 1∶2.5 (red) and (D) an overlaid spectrum between (A) and (C).
(TIF)

Figure S2 **^1^H-^15^N HSQC of ^15^N-labeled** ***holo-me*** **ACP titrated against unlabeled CalE7.** The titration is performed at 25°C till monomeric *holo-me*ACP: CalE7 molar ratio of (A) 1∶0 (black) (B) 1∶1 (blue) (C) 1∶2.5 (red) and (D) an overlaid spectrum between (A) and (C).
(TIF)
