# Supplementary figures and images for: Solution Structures of the Acyl Carrier Protein Domain from the Highly Reducing Type I Iterative Polyketide Synthase CalE8
Source: PLoS One. 2011 Jun 2;6(6):e20549. doi: 10.1371/journal.pone.0020549 (PMC3107222; doi:10.1371/journal.pone.0020549)

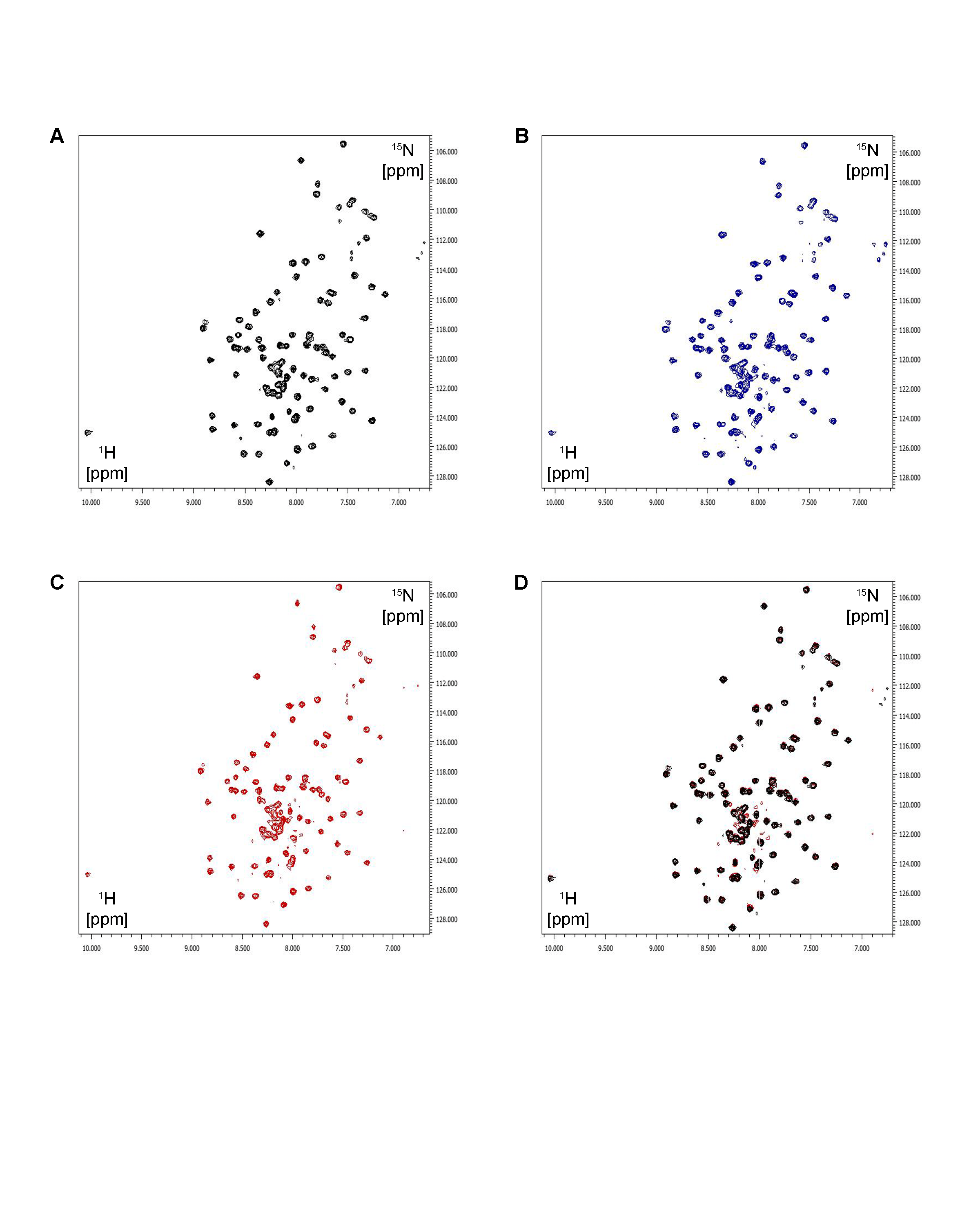

Supplement: Figure S1 — 1H-15N HSQC of 15N-labeled apo-me ACP titrated against unlabeled CalE7. The titration is performed at 25°C in monomeric apo-meACP: CalE7 molar ratio of (A) 1∶0 (black) (B) 1∶1 (blue) (C) 1∶2.5 (red) and (D) an overlaid spectrum between (A) and (C). (TIF) [file pone.0020549.s001.tif]

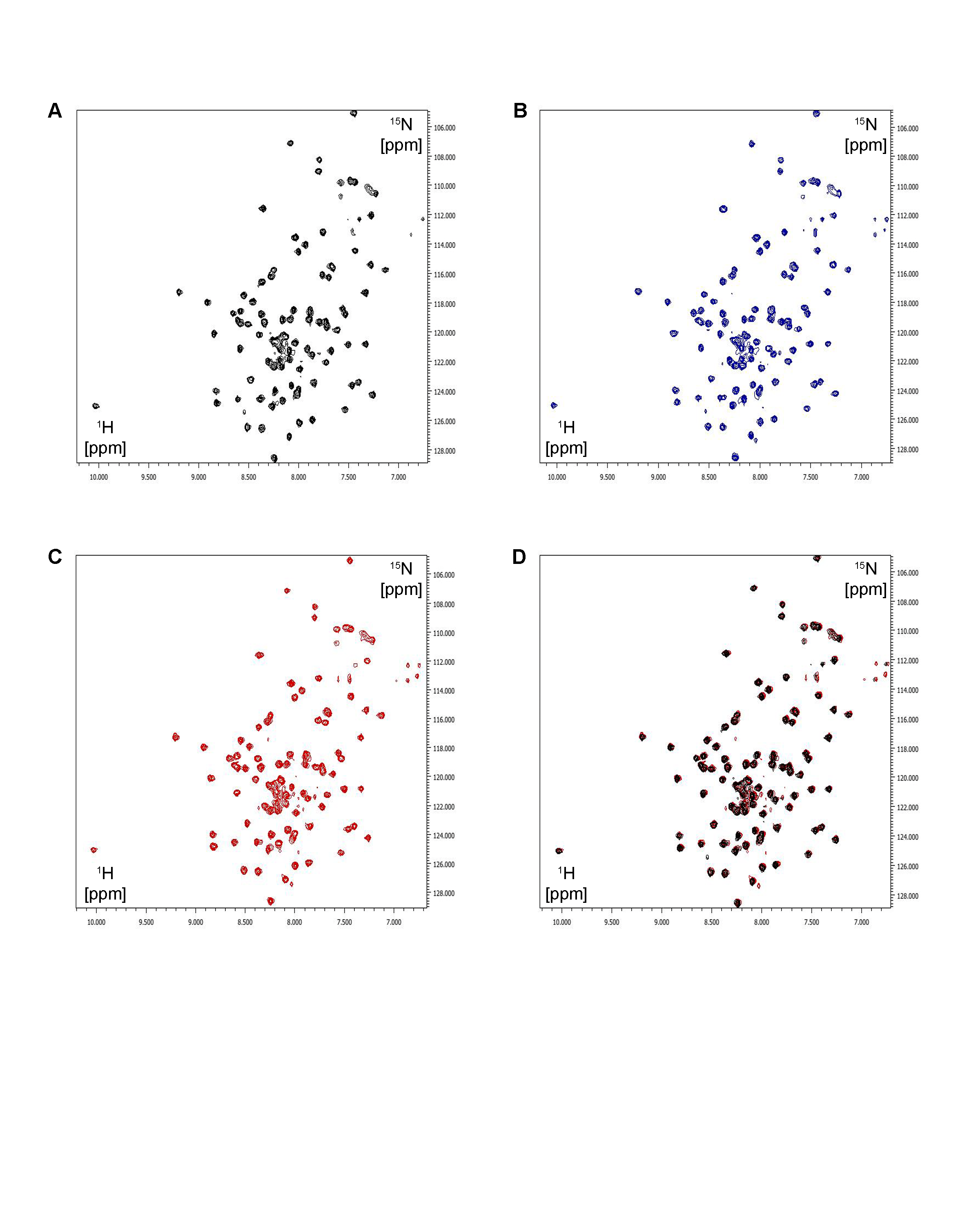

Supplement: Figure S2 — 1H-15N HSQC of 15N-labeled holo-me ACP titrated against unlabeled CalE7. The titration is performed at 25°C till monomeric holo-meACP: CalE7 molar ratio of (A) 1∶0 (black) (B) 1∶1 (blue) (C) 1∶2.5 (red) and (D) an overlaid spectrum between (A) and (C). (TIF) [file pone.0020549.s002.tif]
